# Evaluation of a combined posterior lateral and anteromedial approach in the treatment of terrible triad of the elbow: A retrospective study

**DOI:** 10.1097/MD.0000000000006819

**Published:** 2017-06-02

**Authors:** Hong-Wei Chen, Zi-Yang Wang, Xulin Wu, Bensen Tang, Weimin Zhu, Guangfu Zhou, Fuyao Liu, Bing Qiu

**Affiliations:** Department of Orthopaedics, the Affiliated YiWu Hospital of Wenzhou Medical University, Yiwu, P.R. China.

**Keywords:** a comparative study, anteromedial approach, efficacy evaluation, posterior lateral approach, terrible triad of the elbow

## Abstract

**Background:**

This retrospective study aims to investigate the efficacy and safety of a combined posterior lateral and anteromedial approach in the treatment of terrible triad of the elbow (TTE).

**Methods:**

TTE patients who received a combination of posterior lateral and anteromedial approach or other conservative treatments were included in the present study. The postoperative functions of the elbow and the severity of traumatic arthritis were assessed using the Mayo Elbow Performance Score (MEPS) and visual analog scale (VAS). Extension–flexion of elbow joint and rotation of forearm were also measured.

**Results:**

A combined posterior lateral and anteromedial approach or other conservative treatments showed significant improvements in the activity of the elbow, MEPS, VAS, the excellent rate, and x-ray results. The postoperative healing time and complication rate of patients who received a combined posterior lateral and anteromedial approach significantly decreased compared to those who received other conservative treatments.

**Conclusions:**

Patients with TTE who received a combined posterior lateral and anteromedial treatment had an increased fracture healing rate, showed improved recovery of elbow functions and had fewer complications.

## 1 Introduction

“ Terrible triad of the elbow ” (TTE) consists of elbow dislocation, coronoid process fracture, or radial head fracture.^[1]^ TTE is generally caused by different force vectors which occur during a fall such as rotation of forearm, upper limb ectropion, or the most common “fall on outstretched hand”.^[2]^ Generally, TTE patients suffer from complications such as joint stiffness, ulnar nerve complications, or posttrauma arthritis.^[1,3]^ The main treatments of TTE include conservative or surgical treatments such as the Kocher/lateral or anteromedial approaches.^[4]^ Historically, difficulty in dealing with these injuries has been ascribed to insufficient information regarding elbow stabilization and the lack of appropriate surgical techniques.^[5]^ Therefore, it is important to study the efficacy and safety of the joint KLA and anteromedial in the treatment of TTE.

The KLA approach aims to repair radial head fracture and lateral collateral ligaments.^[6]^ Posterolateral exposure of the elbow is a beneficial technique for addressing complex fractures of the radial head.^[7]^ However, an extensive and deep dissection is generally required in the lateral approach. This may further damage the already traumatized soft tissues in the region and the surgical wound is at a risk of other complications such as deep infection.^[8]^ Additionally, the anteromedial approach facilitates repair of the anterior joint capsular, coronal head, and internal collateral ligamentous injuries.^[9,10]^ When the lateral approach is used in combination with the anteromedial approach, it completely exposes these injuries and can help to restore elbow stability and reconstruct fractures of radial head and lateral ligament complex. This in turn contributes to the restoration and fixation of the coronoid fracture process.^[11]^ Therefore, in this paper, a combined posterior lateral and anteromedial approach and other conservative treatments were compared in the treatment of TTE.

## 2 Materials and method

### 2.1 Ethical statement

The present study was performed in accordance with the guidelines established by the Medicine Ethics Review Committee at the Affiliated YiWu Hospital of Wenzhou Medical University. All patients signed written informed consents.

### 2.2 General information

A total of 44 patients with closed TTE were selected from the Affiliated YiWu Hospital of Wenzhou Medical University from January 2009 to 2014. Thirty-seven cases were randomly chosen for the joint therapy (experiment group) and the remaining 7 cases received conservative treatments consisting of closed reduction and plaster fixation for 8 weeks (control group). The sample contained 25 males and 19 females aged between 33 and 65 years with a mean age of 37.7 ± 4.8 years. There were 21 cases of left-side fractures and 23 cases of right-side fractures. The cause of injury included falls (25 cases) and traffic accidents (19 cases). According to the O’Driscoll classification standard on ulna coracoid process fracture,^[12]^ there were 18 cases of type I fractures, 15 cases of type II fractures, and 11 cases of type III fractures. According to the Mason classification standard on radial head fractures, there were also 12 type I fractures, 21 type II fractures, and 11 type III fractures.^[13]^ The inclusion criteria were as follows: patients conformed to the diagnostic criteria of TTE; patients were over 18 years old and were compliant in terms of treatment and observation; patients could tolerate the surgery (excluding surgical contradictions); patients had completed medical records; and patients had normal functioning of the elbow joint and no prior fractures. The exclusion criteria were as follows: patients suffered from previous fractures or pathologic fractures; patients had metabolic, endocrine or autoimmune fractures; patients suffered from mental illnesses or severe primary diseases in the heart, brain, liver, kidney, or hematopoietic system; and patients had incomplete medical records. Thirty-seven patients in the experiment group underwent surgical treatments 1 to 9 days after injury (average of 5.5 days).

### 2.3 Therapeutic methods

Before surgery, patients underwent preoperative computed tomography (CT) scans to determine the location of fracture displacement. After admission to the hospital, all patients were treated with manual reduction and the elbow joint was supported by external fixation until the situation became stable and suitable for surgery. If required, patients received brachial plexus anesthesia or general anesthesia. After preoperative preparation, 44 patients with closed fractures were operated on 5.5 days (on average) after injury. Antibiotics were taken 20 to 30 minutes before surgery and an x-ray machine was used to monitor the surgery. Patients with type II or type III radial head fractures were treated using the Kirschner wire or cannulated screw fixation. If necessary, artificial radial head replacement was also used in type III fractures. And the avulsion of the complexities of the lateral ulnar collateral ligament from the lateral epicondyle on a stop point must be operative management with the radial head surgery preserved and the connection between the humeroradial joint maintained. The method of coronoid process fracture fixation: a type I nonabsorbable suture was used to fix fracture mass as a base for the coronoid process. For type II or III fractures, the ulna was fixed using 1 or 2 titanium cannulated lag screws from back to front after reduction of postoperative. The joint capsule, lateral collateral ligament and medial accessory ligament was repaired and stability of the elbow joint was examined. If the valgus was unstable before lateral collateral ligament repair, hinged external fixtures were used to assistant and stabilize the bone joint. In the control group, 7 patients under conservative treatments were fixed with closed reduction and plaster for 8 weeks.

### 2.4 Postoperative treatment

After surgery, elbow position of patients was fixed with a bend at 90^o^ using a plaster cast. During the perioperative period (3 days), routine antibiotics were prescribed for infection prevention (IP). During this period, wound care and acral blood circulation was given extra attention. Patients were told to start appropriate exercises for joint movement. Wounds were kept dry and the appropriate pressure and stitches were removed after 2 weeks. Two and 3 weeks after surgery, patients started to do passive exercise such as elbow joint flexion–extension and rotation of the forearm. The plaster cast was removed after 4 weeks and patients started to do active exercises by themselves such as elbow joint flexion–extension and rotation of forearm while avoiding an elbow flexion of above 150^o^ for the first 6 weeks.

### 2.5 Efficacy evaluation

The Mayo Elbow Performance Score (MEPS) was used to evaluate elbow joint stability, activity, and pain of patients.^[14]^ The maximum score is 100 points. A final score of over 90 points was regarded as excellent, 75 to 89 as good, 60 to 74 as acceptable, and a score below 60 as poor. Elbow joint flexion–extension and rotation of the forearm was measured. The fracture site (FS) of patients was observed using an x-ray. Heterotopic ossification (HO) reduction and the complication rate were also recorded.

### 2.6 Follow-up

All the patients received a follow-up check once a month for the first 3 months after surgery. Afterward 3 months, all patients received follow-ups once every 3 months. All 44 patients received follow-up checks for 6 to 15 months after surgery (10 months was the median). The follow-ups included: a lateral x-ray of the elbow joint; elbow joint flexion–extension and rotation of the forearm measurements; and (3) a MEPS.

### 2.7 Statistics analysis

All data were analyzed using SPSS 16.0 software. The median was used as the dividing line for the survival analysis and the average values were compared using *t* tests. Measurement data are expressed as mean ± standard deviation (SD) and the *t* test was used for verification. Categorical data were verified using the *χ*^2^ test. *P* <.05 indicates that a difference is statistically significant.

## 3 Results

### 3.1 Baseline characteristics of the study subjects

The study subjects included 25 males and 19 females aged between 33 and 65 years old, mean age of 37.7 ± 4.8 years. There were 21 cases of left-side fractures and 23 cases of right-side fractures. The causes of fractures included falls (25 cases) and traffic accidents (19 cases). The age, mean age, smoking status, alcohol consumption, cause of fracture, FS, body mass index (BMI), mean duration of injury, and length of stay (LOS) were not statistically different between the experiment and control groups (all *P* >.05, Table 1).

**Table 1:**
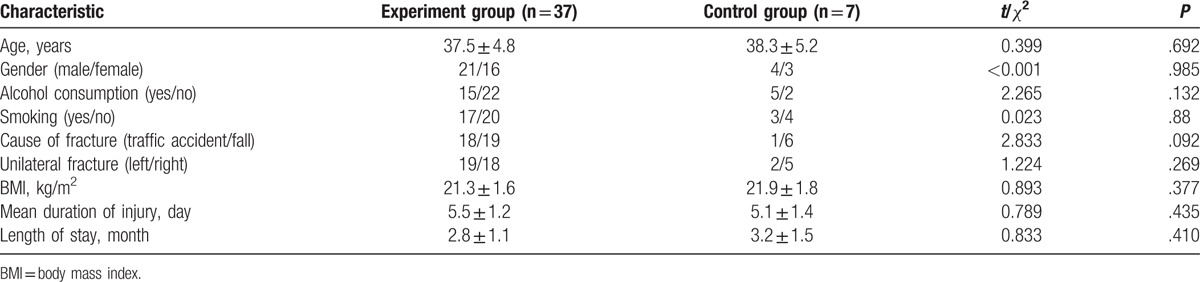
Baseline characteristics of study subjects in the experiment and control groups.

### 3.2 Healing time and complication rates in the experiment and control groups

The healing time and complication rate in the experiment and control groups (Table 2) were statistically significant (both *P* <.05). Complications in this experiment included superficial or deep infection, skin necrosis, and elbow dislocation. Thirty-seven cases had no complications, however, 3 TTE patients who received conservative treatments suffered from elbow pain, discomfort, and limited range of motion.

**Table 2:**
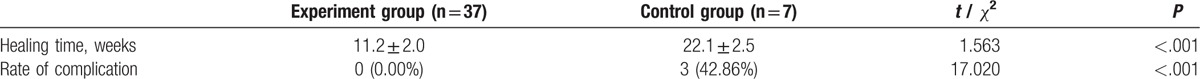
Comparison of the healing time and the rate of complication between the experiment and control groups.

### 3.3 X-ray results in the experiment and control groups

**Figure 1:**
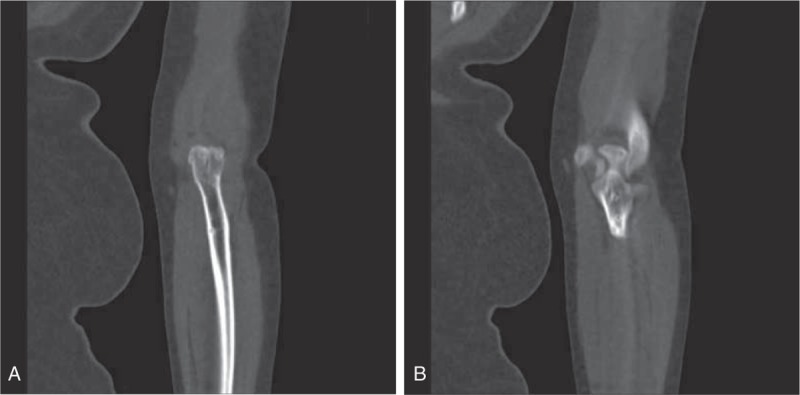
The CT scan firm of fracture site in the experiment and control groups. (A) Patient treated with a posterior lateral approach combined with a anteromedial approach; (B) patients received conservative treatments. CT = computer tomography.

In the experiment group (A), all FSs healed. There was proper fixation, no flexibility in the FS x-ray, and there was no elbow joint dislocation or HO. The CT scan also revealed significant fracture improvements. However, in the control group (B) FSs did not heal as well (the CT scan shows the patients’ fractures in Fig. 1).

### 3.4 MEPS efficacy evaluation

The MEPS was utilized to evaluate elbow joint stability, activity, and pain of patients. In the experiment group, the results gave scores with a range of 75 and 98 and an average score of 89.9. A total of 17 cases were considered excellent (representing 75.7% of results), 11 cases considered good, 7 cases considered average, and 2 cases considered poor. In the control group, 4 cases were considered poor and 3 cases were considered average. Of the 7 patients in the control group who received conservative treatments (MEPS, VAS), the rate of cases that were considered excellent was statistically different to the experiment group (all *P* <.05, Table 3).

**Table 3:**
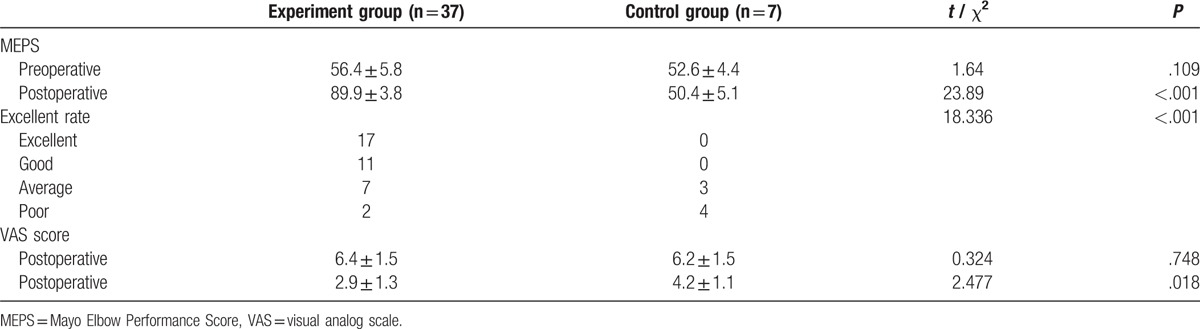
Comparisons of the MEPS, VAS, and the excellent rate between the experiment and control groups.

### 3.5 Comparison of postoperative elbow functioning of the experiment and control groups

After a combined posterior lateral and anteromedial approach in the treatment of TTE, elbow joint flexion–extension and forearm rotation was significantly improved in the experiment and control groups (both *P* <.05). In the experiment group before treatment, the average elbow joint flexion was between 85° and 125°, and after treatment it was 116.5°. Before treatment, the average rotation of the forearm was between 95° and 140°, and after treatment it was 125.5°. In the control group before treatment, the elbow joint flexion was 70° to 95°, and after treatment it was 79.5°. Before treatment, the average rotation of the forearm was between 80^o^ and 110°, and after treatment it was 105.5°. There is a statistically significant difference between the before and after surgery results of the two groups (both *P* <.05, Table 4).

**Table 4:**
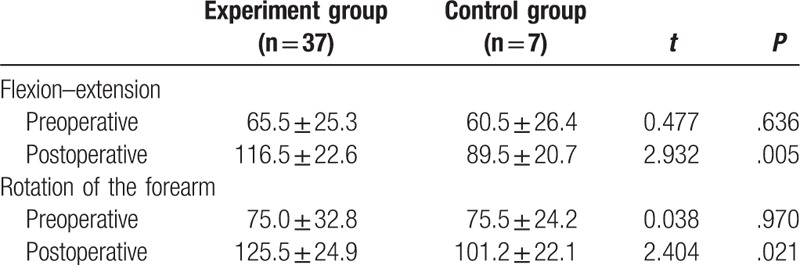
Comparison of the activity of elbow joint between the experiment and control groups.

## 4 Discussion

TTE can be caused by high-energy trauma during a fall, which may also lead to serious elbow instability. Early surgical intervention is paramount in avoiding complications.^[4]^ Although conservative treatments can deal with simple elbow dislocations, a more complex elbow fracture or dislocation requires a more intensive treatment.^[15]^ Therefore, it is imperative to find a reasonable and effective surgical treatment for TTE.

In this study, a combined posterior lateral and anteromedial approach had significant benefits in regards to healing time and complication rate over traditional conservative methods. Using a combined posterior lateral and anteromedial approach, patients showed no elbow dislocation or other complications. Furthermore, the CT results further confirmed the later rehabilitation improvements. Additionally, patients who were treated with the conservative methods showed significantly limited elbow activity and discomfort. During the surgical treatment period, elbow stability should be restored through brachioradialis connection, lateral collateral ligament repair, or internal fixation of the coronal position.^[16]^ A combined posterior lateral and anteromedial approach provides sufficient operation space and full exposure of the wound to facilitate the effective repair of soft tissue injury and bone structure fixation damages. In this study, all the elbow FSs achieved solid coalescence after the operation without delay, absent coalescence or complications. Many patients regained elbow stability even after suffering serious TTE injuries. The efficacy of conservative treatments however is quite poor, as demonstrated by the prolonged healing time and increased rate of complications.

It was also found in this study that elbow functioning scores were higher in patients who received a combined posterior lateral and anteromedial approach than in those who received conservative treatments. Interestingly, Kälicke et al^[17]^ demonstrated that a prerequisite for good elbow function is to replicate exercises, which helps to stabilize the elbow joint and reconstruct the coronoid process. Apart from a smaller incision in the lateral approach, it provides better exposure for radial head fracture fixation and the repair of the lateral collateral ligament than the posterior approach.^[18]^ The anteromedial approach also has a clear exposure of ulnar coronoid process and radial head fractures. This can anatomically restore serious types II and III fractures, achieve good elbow stability, and improve functioning.^[19]^ Moreover, this study shows that the Mayo score in joint operation is significantly improved compared to the conservative approach. de Aquino Santos et al^[20]^ also demonstrated that when functional activity of the elbow is taken into consideration, the results of the surgery were usually satisfactory. This is consistent with our results.

This study aimed to investigate the clinical efficacy and safety of a joint posterior lateral and anteromedial approach in TTE treatment. It was found that the joint approach yielded higher healing rates, fewer complication rates, and is worthy of being promoted for clinical applications. However, because of the complex mechanisms involved in the pathogenesis of TTE, follow-up studies are required.
