# Evaluation of a combined posterior lateral and anteromedial approach in the treatment of terrible triad of the elbow: A retrospective study: Erratum

**DOI:** 10.1097/MD.0000000000007313

**Published:** 2017-06-16

In the article, “Evaluation of a combined posterior lateral and anteromedial approach in the treatment of terrible triad of the elbow: A retrospective study”,^[1]^ which appeared in Volume 96, Issue 22 of *Medicine*, the author byline should have appeared as “Xulin Wu, MM, Bensen Tang, BA, Weimin Zhu, BA, Guangfu Zhou, BA, Fuyao Liu, BA, Bing Qiu^∗^, BA, Hong-Wei Chen, MB, Zi-Yang Wang, MB.” The corresponding author's information should have appeared as “Bing Qiu, Department of Orthopedics,Guizhou Province Orthopedics Hospital,123 Shachong S Rd,Guiyang 550007, China. Email: yz8088gk@163.com, bingqiu_1@126.com. Tel: +86 0851-85792149; Fax: +86 0851-85772911.”
