# Evaluation of a combined posterior lateral and anteromedial approach in the treatment of terrible triad of the elbow: A retrospective study: Erratum

**DOI:** 10.1097/MD.0000000000007643

**Published:** 2017-07-21

In the article, “Evaluation of a combined posterior lateral and anteromedial approach in the treatment of terrible triad of the elbow: A retrospective study”,^[1]^ which appeared in Volume 96, Issue 24 of *Medicine*, in the following sentences, “Affiliated YiWu Hospital of Wenzhou Medical University” should have appeared as “Guizhou Province Orthopedics Hospital.”

“The present study was performed in accordance with the guidelines established by the Medicine Ethics Review Committee at the Affiliated YiWu Hospital of Wenzhou Medical University.”

“A total of 44 patients with closed TTE were selected from the Affiliated YiWu Hospital of Wenzhou Medical University from January 2009 to 2014.”
