# Evaluation of a combined posterior lateral and anteromedial approach in the treatment of terrible triad of the elbow: A retrospective study: Erratum

**DOI:** 10.1097/MD.0000000000007827

**Published:** 2017-08-11

In the erratum for “Evaluation of a combined posterior lateral and anteromedial approach in the treatment of terrible triad of the elbow: A retrospective study”,^[1]^ which appeared in Volume 96, Issue 29 of *Medicine*, the erratum linked to another erratum and contained the reference to this erratum. The reference should have appeared as “Chen H-W, Wang Z-Y, Wu X. Evaluation of a combined posterior lateral and anteromedial approach in the treatment of terrible triad of the elbow: A retrospective study. *Medicine*. 96;22:e6819.” and has since been changed in the original and the link corrected.
